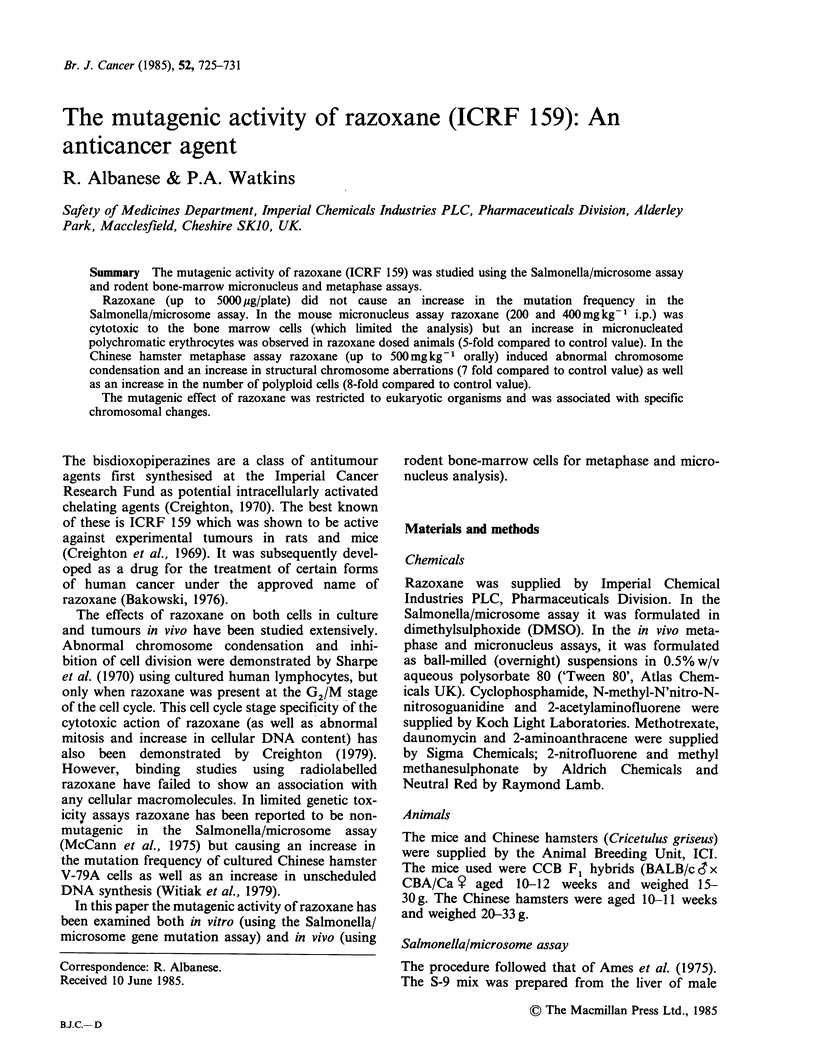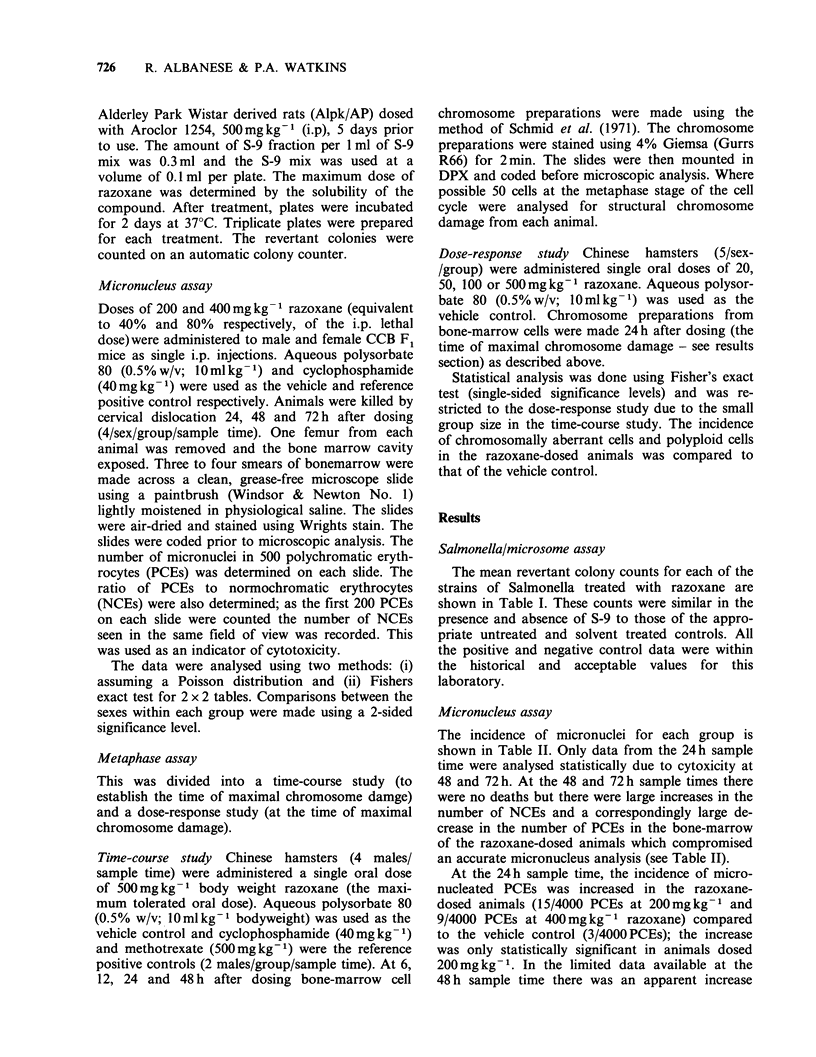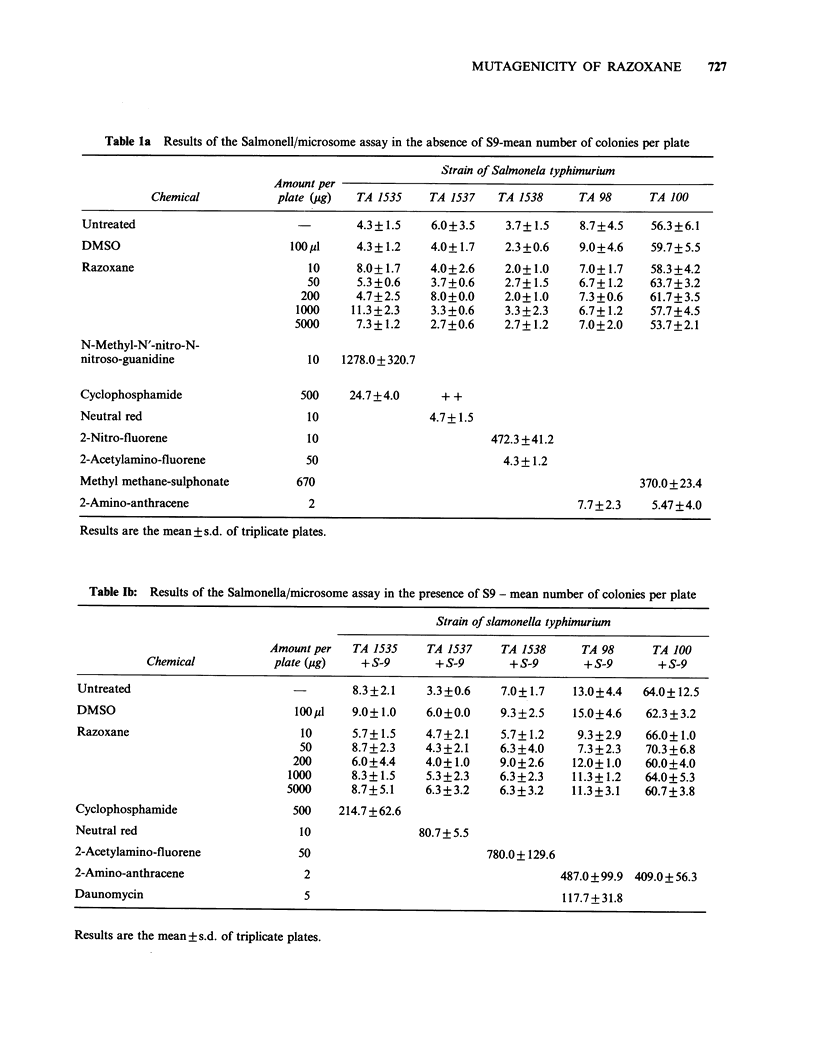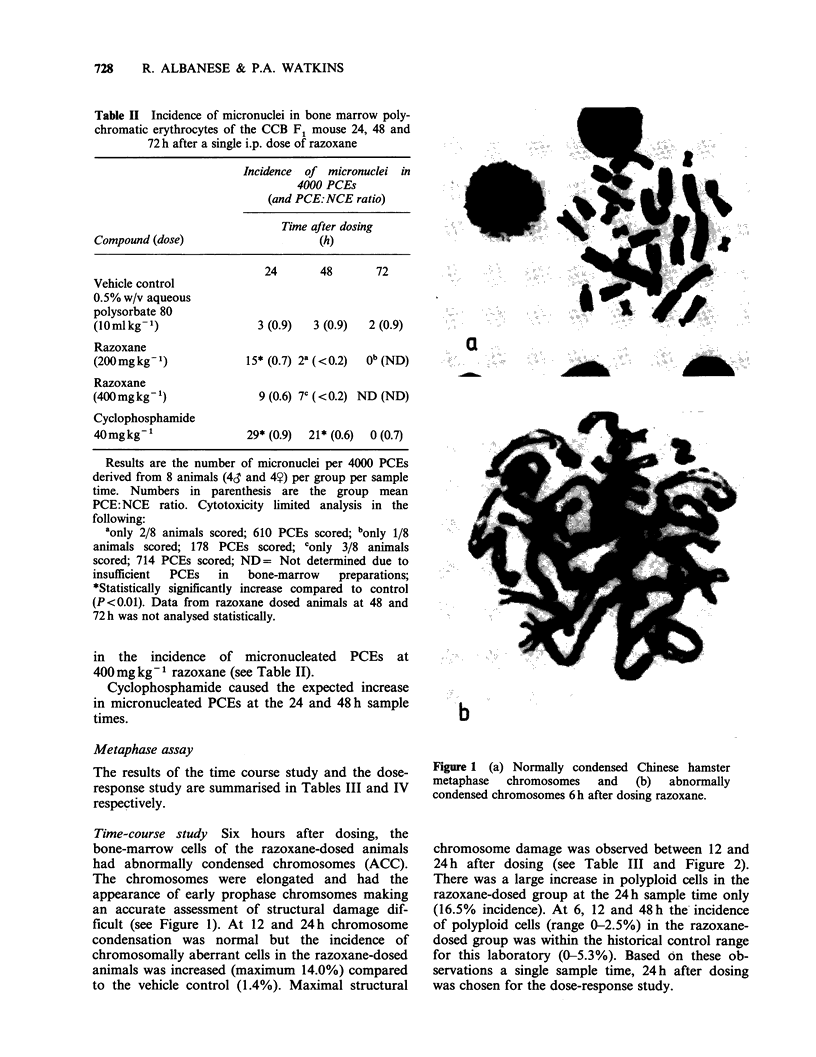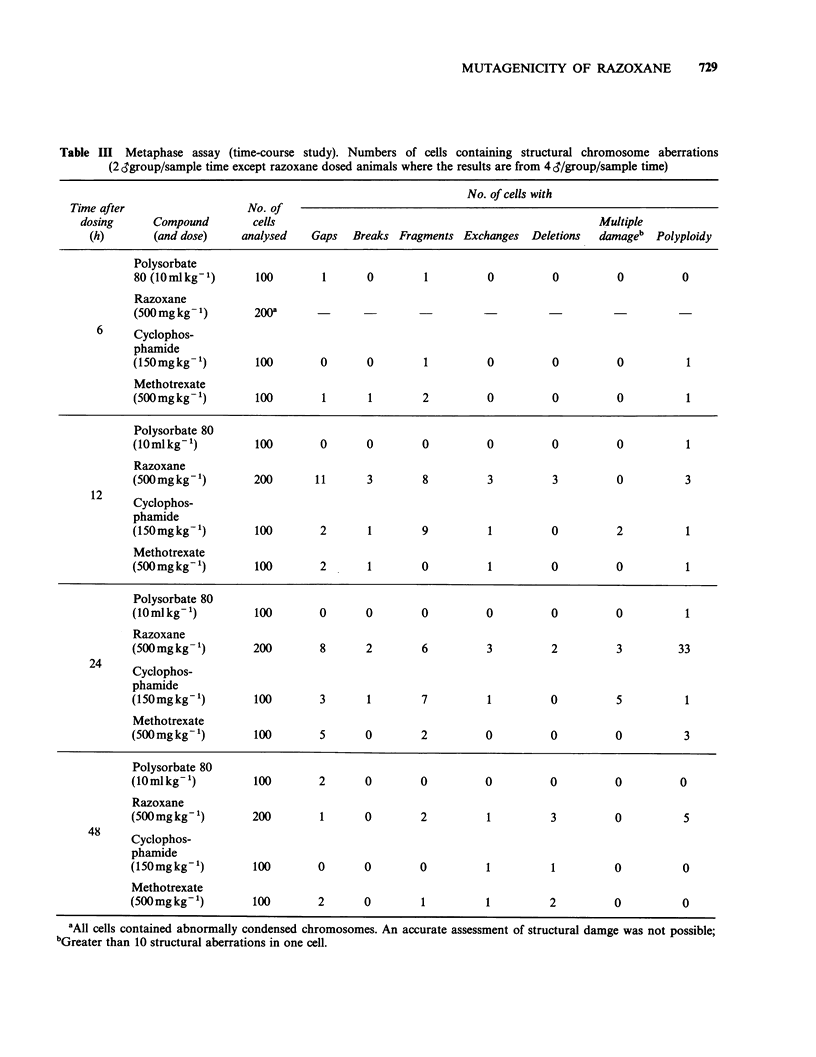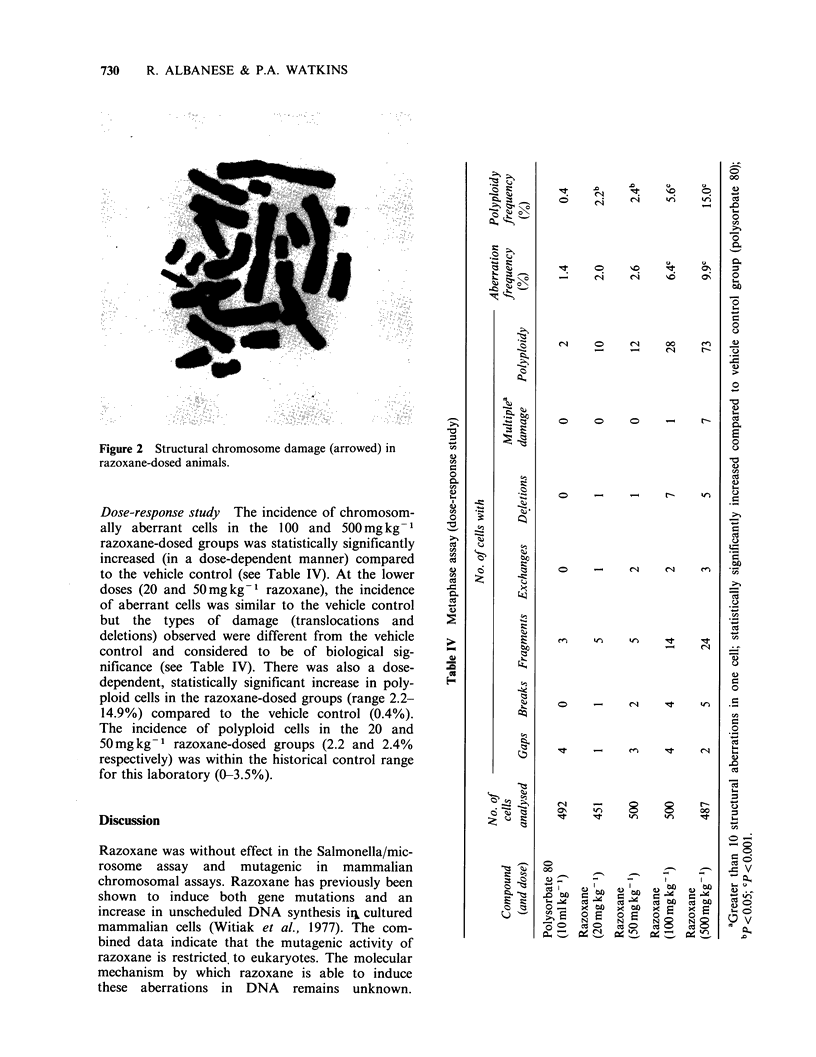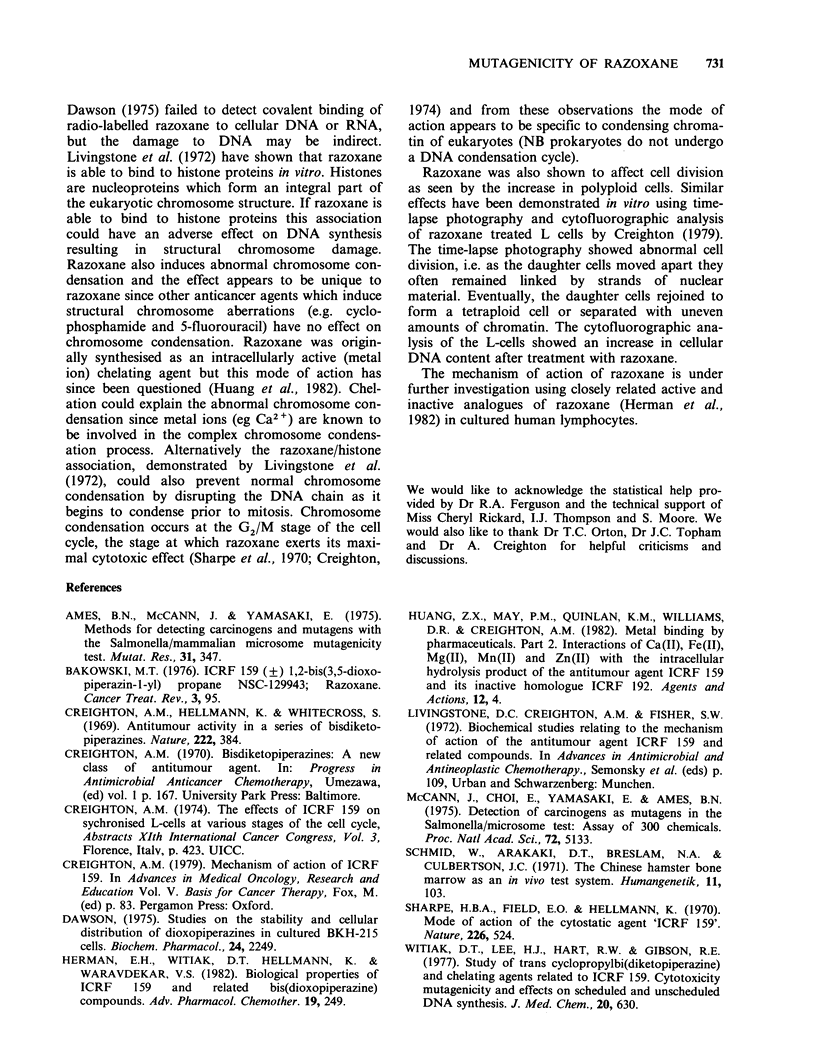# The mutagenic activity of razoxane (ICRF 159): an anticancer agent.

**DOI:** 10.1038/bjc.1985.250

**Published:** 1985-11

**Authors:** R. Albanese, P. A. Watkins

## Abstract

**Images:**


*Br. J. Cancer* (1985), **52**, 725–731

### The mutagenic activity of razoxane (ICRF 159): An anticancer agent

R. Albanese & P.A. Watkins

*Safety of Medicines Department, Imperial Chemicals Industries PLC, Pharmaceuticals Division, Alderley Park, Macclesfield, Cheshire SK10, UK.*

**Summary** The mutagenic activity of razoxane (ICRF 159) was studied using the Salmonella/microsome assay and rodent bone-marrow micronucleus and metaphase assays.

Razoxane (up to 5000 $\mu$g/plate) did not cause an increase in the mutation frequency in the Salmonella/microsome assay. In the mouse micronucleus assay razoxane (200 and 400 mg kg$^{-1}$ i.p.) was cytotoxic to the bone marrow cells (which limited the analysis) but an increase in micronucleated polychromatic erythrocytes was observed in razoxane dosed animals (5-fold compared to control value). In the Chinese hamster metaphase assay razoxane (up to 500 mg kg$^{-1}$ orally) induced abnormal chromosome condensation and an increase in structural chromosome aberrations (7 fold compared to control value) as well as an increase in the number of polyploid cells (8-fold compared to control value).

The mutagenic effect of razoxane was restricted to eukaryotic organisms and was associated with specific chromosomal changes.

The bisdioxopiperazines are a class of antitumour agents first synthesised at the Imperial Cancer Research Fund as potential intracellularly activated chelating agents (Creighton, 1970). The best known of these is ICRF 159 which was shown to be active against experimental tumours in rats and mice (Creighton *et al.*, 1969). It was subsequently developed as a drug for the treatment of certain forms of human cancer under the approved name of razoxane (Bakowski, 1976).

The effects of razoxane on both cells in culture and tumours *in vivo* have been studied extensively. Abnormal chromosome condensation and inhibition of cell division were demonstrated by Sharpe *et al.* (1970) using cultured human lymphocytes, but only when razoxane was present at the $G_2$/M stage of the cell cycle. This cell cycle stage specificity of the cytotoxic action of razoxane (as well as abnormal mitosis and increase in cellular DNA content) has also been demonstrated by Creighton (1979). However, binding studies using radiolabelled razoxane have failed to show an association with any cellular macromolecules. In limited genetic toxicity assays razoxane has been reported to be non-mutagenic in the Salmonella/microsome assay (McCann *et al.*, 1975) but causing an increase in the mutation frequency of cultured Chinese hamster V-79A cells as well as an increase in unscheduled DNA synthesis (Witiak *et al.*, 1979).

In this paper the mutagenic activity of razoxane has been examined both *in vitro* (using the Salmonella/microsome gene mutation assay) and *in vivo* (using rodent bone-marrow cells for metaphase and micronucleus analysis).

Correspondence: R. Albanese.
Received 10 June 1985.

### Materials and methods

### *Chemicals*

Razoxane was supplied by Imperial Chemical Industries PLC, Pharmaceuticals Division. In the Salmonella/microsome assay it was formulated in dimethylsulphoxide (DMSO). In the *in vivo* metaphase and micronucleus assays, it was formulated as ball-milled (overnight) suspensions in 0.5% w/v aqueous polysorbate 80 ('Tween 80', Atlas Chemicals UK). Cyclophosphamide, N-methyl-N'nitro-N-nitrosoguanidine and 2-acetylaminofluorene were supplied by Koch Light Laboratories. Methotrexate, daunomycin and 2-aminoanthracene were supplied by Sigma Chemicals; 2-nitrofluorene and methyl methanesulphonate by Aldrich Chemicals and Neutral Red by Raymond Lamb.

### *Animals*

The mice and Chinese hamsters (*Cricetulus griseus*) were supplied by the Animal Breeding Unit, ICI. The mice used were CCB $F_1$ hybrids (BALB/c ♂ × CBA/Ca ♀ aged 10–12 weeks and weighed 15–30 g. The Chinese hamsters were aged 10–11 weeks and weighed 20–33 g.

### *Salmonella/microsome assay*

The procedure followed that of Ames *et al.* (1975). The S-9 mix was prepared from the liver of male

© The Macmillan Press Ltd., 1985

Alderley Park Wistar derived rats (Alpk/AP) dosed with Aroclor 1254, 500 mg kg$^{-1}$ (i.p), 5 days prior to use. The amount of S-9 fraction per 1 ml of S-9 mix was 0.3 ml and the S-9 mix was used at a volume of 0.1 ml per plate. The maximum dose of razoxane was determined by the solubility of the compound. After treatment, plates were incubated for 2 days at 37°C. Triplicate plates were prepared for each treatment. The revertant colonies were counted on an automatic colony counter.

*Micronucleus assay*

Doses of 200 and 400 mg kg$^{-1}$ razoxane (equivalent to 40% and 80% respectively, of the i.p. lethal dose) were administered to male and female CCB $F_1$ mice as single i.p. injections. Aqueous polysorbate 80 (0.5% w/v; 10 ml kg$^{-1}$) and cyclophosphamide (40 mg kg$^{-1}$) were used as the vehicle and reference positive control respectively. Animals were killed by cervical dislocation 24, 48 and 72 h after dosing (4/sex/group/sample time). One femur from each animal was removed and the bone marrow cavity exposed. Three to four smears of bonemarrow were made across a clean, grease-free microscope slide using a paintbrush (Windsor & Newton No. 1) lightly moistened in physiological saline. The slides were air-dried and stained using Wrights stain. The slides were coded prior to microscopic analysis. The number of micronuclei in 500 polychromatic erythrocytes (PCEs) was determined on each slide. The ratio of PCEs to normochromatic erythrocytes (NCEs) were also determined; as the first 200 PCEs on each slide were counted the number of NCEs seen in the same field of view was recorded. This was used as an indicator of cytotoxicity.

The data were analysed using two methods: (i) assuming a Poisson distribution and (ii) Fishers exact test for 2 x 2 tables. Comparisons between the sexes within each group were made using a 2-sided significance level.

*Metaphase assay*

This was divided into a time-course study (to establish the time of maximal chromosome damge) and a dose-response study (at the time of maximal chromosome damage).

*Time-course study* Chinese hamsters (4 males/sample time) were administered a single oral dose of 500 mg kg$^{-1}$ body weight razoxane (the maximum tolerated oral dose). Aqueous polysorbate 80 (0.5% w/v; 10 ml kg$^{-1}$ bodyweight) was used as the vehicle control and cyclophosphamide (40 mg kg$^{-1}$) and methotrexate (500 mg kg$^{-1}$) were the reference positive controls (2 males/group/sample time). At 6, 12, 24 and 48 h after dosing bone-marrow cell chromosome preparations were made using the method of Schmid *et al.* (1971). The chromosome preparations were stained using 4% Giemsa (Gurrs R66) for 2 min. The slides were then mounted in DPX and coded before microscopic analysis. Where possible 50 cells at the metaphase stage of the cell cycle were analysed for structural chromosome damage from each animal.

*Dose-response study* Chinese hamsters (5/sex-/group) were administered single oral doses of 20, 50, 100 or 500 mg kg$^{-1}$ razoxane. Aqueous polysorbate 80 (0.5% w/v; 10 ml kg$^{-1}$) was used as the vehicle control. Chromosome preparations from bone-marrow cells were made 24 h after dosing (the time of maximal chromosome damage – see results section) as described above.

Statistical analysis was done using Fisher's exact test (single-sided significance levels) and was restricted to the dose-response study due to the small group size in the time-course study. The incidence of chromosomally aberrant cells and polyploid cells in the razoxane-dosed animals was compared to that of the vehicle control.

### Results

*Salmonella/microsome assay*

The mean revertant colony counts for each of the strains of Salmonella treated with razoxane are shown in Table I. These counts were similar in the presence and absence of S-9 to those of the appropriate untreated and solvent treated controls. All the positive and negative control data were within the historical and acceptable values for this laboratory.

*Micronucleus assay*

The incidence of micronuclei for each group is shown in Table II. Only data from the 24 h sample time were analysed statistically due to cytoxicity at 48 and 72 h. At the 48 and 72 h sample times there were no deaths but there were large increases in the number of NCEs and a correspondingly large decrease in the number of PCEs in the bone-marrow of the razoxane-dosed animals which compromised an accurate micronucleus analysis (see Table II).

At the 24 h sample time, the incidence of micronucleated PCEs was increased in the razoxane-dosed animals (15/4000 PCEs at 200 mg kg$^{-1}$ and 9/4000 PCEs at 400 mg kg$^{-1}$ razoxane) compared to the vehicle control (3/4000 PCEs); the increase was only statistically significant in animals dosed 200 mg kg$^{-1}$. In the limited data available at the 48 h sample time there was an apparent increase

**Table 1a** Results of the Salmonell/microsome assay in the absence of S9-mean number of colonies per plate

| Chemical | Amount per plate (μg) | *Strain of Salmonela typhimurium* TA 1535 | TA 1537 | TA 1538 | TA 98 | TA 100 |
|---|---|---|---|---|---|---|
| Untreated | — | 4.3±1.5 | 6.0±3.5 | 3.7±1.5 | 8.7±4.5 | 56.3±6.1 |
| DMSO | 100 μl | 4.3±1.2 | 4.0±1.7 | 2.3±0.6 | 9.0±4.6 | 59.7±5.5 |
| Razoxane | 10 | 8.0±1.7 | 4.0±2.6 | 2.0±1.0 | 7.0±1.7 | 58.3±4.2 |
| | 50 | 5.3±0.6 | 3.7±0.6 | 2.7±1.5 | 6.7±1.2 | 63.7±3.2 |
| | 200 | 4.7±2.5 | 8.0±0.0 | 2.0±1.0 | 7.3±0.6 | 61.7±3.5 |
| | 1000 | 11.3±2.3 | 3.3±0.6 | 3.3±2.3 | 6.7±1.2 | 57.7±4.5 |
| | 5000 | 7.3±1.2 | 2.7±0.6 | 2.7±1.2 | 7.0±2.0 | 53.7±2.1 |
| N-Methyl-N′-nitro-N-nitroso-guanidine | 10 | 1278.0±320.7 | | | | |
| Cyclophosphamide | 500 | 24.7±4.0 | ++ | | | |
| Neutral red | 10 | | 4.7±1.5 | | | |
| 2-Nitro-fluorene | 10 | | | 472.3±41.2 | | |
| 2-Acetylamino-fluorene | 50 | | | 4.3±1.2 | | |
| Methyl methane-sulphonate | 670 | | | | | 370.0±23.4 |
| 2-Amino-anthracene | 2 | | | | 7.7±2.3 | 5.47±4.0 |

Results are the mean±s.d. of triplicate plates.

**Table Ib:** Results of the Salmonella/microsome assay in the presence of S9 – mean number of colonies per plate

| Chemical | Amount per plate (μg) | *Strain of slamonella typhimurium* TA 1535 +S-9 | TA 1537 +S-9 | TA 1538 +S-9 | TA 98 +S-9 | TA 100 +S-9 |
|---|---|---|---|---|---|---|
| Untreated | — | 8.3±2.1 | 3.3±0.6 | 7.0±1.7 | 13.0±4.4 | 64.0±12.5 |
| DMSO | 100 μl | 9.0±1.0 | 6.0±0.0 | 9.3±2.5 | 15.0±4.6 | 62.3±3.2 |
| Razoxane | 10 | 5.7±1.5 | 4.7±2.1 | 5.7±1.2 | 9.3±2.9 | 66.0±1.0 |
| | 50 | 8.7±2.3 | 4.3±2.1 | 6.3±4.0 | 7.3±2.3 | 70.3±6.8 |
| | 200 | 6.0±4.4 | 4.0±1.0 | 9.0±2.6 | 12.0±1.0 | 60.0±4.0 |
| | 1000 | 8.3±1.5 | 5.3±2.3 | 6.3±2.3 | 11.3±1.2 | 64.0±5.3 |
| | 5000 | 8.7±5.1 | 6.3±3.2 | 6.3±3.2 | 11.3±3.1 | 60.7±3.8 |
| Cyclophosphamide | 500 | 214.7±62.6 | | | | |
| Neutral red | 10 | | 80.7±5.5 | | | |
| 2-Acetylamino-fluorene | 50 | | | 780.0±129.6 | | |
| 2-Amino-anthracene | 2 | | | | 487.0±99.9 | 409.0±56.3 |
| Daunomycin | 5 | | | | 117.7±31.8 | |

Results are the mean±s.d. of triplicate plates.

**Table II** Incidence of micronuclei in bone marrow polychromatic erythrocytes of the CCB $F_1$ mouse 24, 48 and 72 h after a single i.p. dose of razoxane

| *Compound (dose)* | *Incidence of micronuclei in 4000 PCEs (and PCE:NCE ratio)* | | |
|---|---|---|---|
| | *Time after dosing (h)* | | |
| | 24 | 48 | 72 |
| Vehicle control 0.5% w/v aqueous polysorbate 80 ($10\,ml\,kg^{-1}$) | 3 (0.9) | 3 (0.9) | 2 (0.9) |
| Razoxane ($200\,mg\,kg^{-1}$) | 15* (0.7) | 2[a] (<0.2) | 0[b] (ND) |
| Razoxane ($400\,mg\,kg^{-1}$) | 9 (0.6) | 7[c] (<0.2) | ND (ND) |
| Cyclophosphamide $40\,mg\,kg^{-1}$ | 29* (0.9) | 21* (0.6) | 0 (0.7) |

Results are the number of micronuclei per 4000 PCEs derived from 8 animals (4♂ and 4♀) per group per sample time. Numbers in parenthesis are the group mean PCE:NCE ratio. Cytotoxicity limited analysis in the following:
[a]only 2/8 animals scored; 610 PCEs scored; [b]only 1/8 animals scored; 178 PCEs scored; [c]only 3/8 animals scored; 714 PCEs scored; ND = Not determined due to insufficient PCEs in bone-marrow preparations; *Statistically significantly increase compared to control ($P<0.01$). Data from razoxane dosed animals at 48 and 72 h was not analysed statistically.

in the incidence of micronucleated PCEs at $400\,mg\,kg^{-1}$ razoxane (see Table II).

Cyclophosphamide caused the expected increase in micronucleated PCEs at the 24 and 48 h sample times.

*Metaphase assay*

The results of the time course study and the dose-response study are summarised in Tables III and IV respectively.

*Time-course study* Six hours after dosing, the bone-marrow cells of the razoxane-dosed animals had abnormally condensed chromosomes (ACC). The chromosomes were elongated and had the appearance of early prophase chromsomes making an accurate assessment of structural damage difficult (see Figure 1). At 12 and 24 h chromosome condensation was normal but the incidence of chromosomally aberrant cells in the razoxane-dosed animals was increased (maximum 14.0%) compared to the vehicle control (1.4%). Maximal structural chromosome damage was observed between 12 and 24 h after dosing (see Table III and Figure 2). There was a large increase in polyploid cells in the razoxane-dosed group at the 24 h sample time only (16.5% incidence). At 6, 12 and 48 h the incidence of polyploid cells (range 0–2.5%) in the razoxane-dosed group was within the historical control range for this laboratory (0–5.3%). Based on these observations a single sample time, 24 h after dosing was chosen for the dose-response study.

**Figure 1** (a) Normally condensed Chinese hamster metaphase chromosomes and (b) abnormally condensed chromosomes 6 h after dosing razoxane.

**Table III** Metaphase assay (time-course study). Numbers of cells containing structural chromosome aberrations (2 ♂group/sample time except razoxane dosed animals where the results are from 4 ♂/group/sample time)

| Time after dosing (*h*) | Compound (*and dose*) | No. of cells analysed | No. of cells with Gaps | Breaks | Fragments | Exchanges | Deletions | Multiple damage[b] | Polyploidy |
|---|---|---|---|---|---|---|---|---|---|
| 6 | Polysorbate 80 (10 ml kg$^{-1}$) | 100 | 1 | 0 | 1 | 0 | 0 | 0 | 0 |
| | Razoxane (500 mg kg$^{-1}$) | 200[a] | — | — | — | — | — | — | — |
| | Cyclophos-phamide (150 mg kg$^{-1}$) | 100 | 0 | 0 | 1 | 0 | 0 | 0 | 1 |
| | Methotrexate (500 mg kg$^{-1}$) | 100 | 1 | 1 | 2 | 0 | 0 | 0 | 1 |
| 12 | Polysorbate 80 (10 ml kg$^{-1}$) | 100 | 0 | 0 | 0 | 0 | 0 | 0 | 1 |
| | Razoxane (500 mg kg$^{-1}$) | 200 | 11 | 3 | 8 | 3 | 3 | 0 | 3 |
| | Cyclophos-phamide (150 mg kg$^{-1}$) | 100 | 2 | 1 | 9 | 1 | 0 | 2 | 1 |
| | Methotrexate (500 mg kg$^{-1}$) | 100 | 2 | 1 | 0 | 1 | 0 | 0 | 1 |
| 24 | Polysorbate 80 (10 ml kg$^{-1}$) | 100 | 0 | 0 | 0 | 0 | 0 | 0 | 1 |
| | Razoxane (500 mg kg$^{-1}$) | 200 | 8 | 2 | 6 | 3 | 2 | 3 | 33 |
| | Cyclophos-phamide (150 mg kg$^{-1}$) | 100 | 3 | 1 | 7 | 1 | 0 | 5 | 1 |
| | Methotrexate (500 mg kg$^{-1}$) | 100 | 5 | 0 | 2 | 0 | 0 | 0 | 3 |
| 48 | Polysorbate 80 (10 ml kg$^{-1}$) | 100 | 2 | 0 | 0 | 0 | 0 | 0 | 0 |
| | Razoxane (500 mg kg$^{-1}$) | 200 | 1 | 0 | 2 | 1 | 3 | 0 | 5 |
| | Cyclophos-phamide (150 mg kg$^{-1}$) | 100 | 0 | 0 | 0 | 1 | 1 | 0 | 0 |
| | Methotrexate (500 mg kg$^{-1}$) | 100 | 2 | 0 | 1 | 1 | 2 | 0 | 0 |

[a]All cells contained abnormally condensed chromosomes. An accurate assessment of structural damge was not possible; [b]Greater than 10 structural aberrations in one cell.

**Figure 2** Structural chromosome damage (arrowed) in razoxane-dosed animals.

*Dose-response study* The incidence of chromosomally aberrant cells in the 100 and 500 mg kg$^{-1}$ razoxane-dosed groups was statistically significantly increased (in a dose-dependent manner) compared to the vehicle control (see Table IV). At the lower doses (20 and 50 mg kg$^{-1}$ razoxane), the incidence of aberrant cells was similar to the vehicle control but the types of damage (translocations and deletions) observed were different from the vehicle control and considered to be of biological significance (see Table IV). There was also a dose-dependent, statistically significant increase in polyploid cells in the razoxane-dosed groups (range 2.2–14.9%) compared to the vehicle control (0.4%). The incidence of polyploid cells in the 20 and 50 mg kg$^{-1}$ razoxane-dosed groups (2.2 and 2.4% respectively) was within the historical control range for this laboratory (0–3.5%).

### Discussion

Razoxane was without effect in the Salmonella/microsome assay and mutagenic in mammalian chromosomal assays. Razoxane has previously been shown to induce both gene mutations and an increase in unscheduled DNA synthesis in cultured mammalian cells (Witiak *et al.*, 1977). The combined data indicate that the mutagenic activity of razoxane is restricted to eukaryotes. The molecular mechanism by which razoxane is able to induce these aberrations in DNA remains unknown.

**Table IV** Metaphase assay (dose-response study)

| *Compound (and dose)* | *No. of cells analysed* | No. of cells with: *Gaps* | *Breaks* | *Fragments* | *Exchanges* | *Deletions* | *Multiple[a] damage* | *Polyploidy* | *Aberration frequency (%)* | *Polyploidy frequency (%)* |
|---|---|---|---|---|---|---|---|---|---|---|
| Polysorbate 80 (10 ml kg$^{-1}$) | 492 | 4 | 0 | 3 | 0 | 0 | 0 | 2 | 1.4 | 0.4 |
| Razoxane (20 mg kg$^{-1}$) | 451 | 1 | 1 | 5 | 1 | 1 | 0 | 10 | 2.0 | 2.2[b] |
| Razoxane (50 mg kg$^{-1}$) | 500 | 3 | 2 | 5 | 2 | 1 | 0 | 12 | 2.6 | 2.4[b] |
| Razoxane (100 mg kg$^{-1}$) | 500 | 4 | 4 | 14 | 2 | 7 | 1 | 28 | 6.4[c] | 5.6[c] |
| Razoxane (500 mg kg$^{-1}$) | 487 | 2 | 5 | 24 | 3 | 5 | 7 | 73 | 9.9[c] | 15.0[c] |

[a]Greater than 10 structural aberrations in one cell; statistically significantly increased compared to vehicle control group (polysorbate 80); [b]$P < 0.05$; [c]$P < 0.001$.

Dawson (1975) failed to detect covalent binding of radio-labelled razoxane to cellular DNA or RNA, but the damage to DNA may be indirect. Livingstone *et al.* (1972) have shown that razoxane is able to bind to histone proteins *in vitro*. Histones are nucleoproteins which form an integral part of the eukaryotic chromosome structure. If razoxane is able to bind to histone proteins this association could have an adverse effect on DNA synthesis resulting in structural chromosome damage. Razoxane also induces abnormal chromosome condensation and the effect appears to be unique to razoxane since other anticancer agents which induce structural chromosome aberrations (e.g. cyclophosphamide and 5-fluorouracil) have no effect on chromosome condensation. Razoxane was originally synthesised as an intracellularly active (metal ion) chelating agent but this mode of action has since been questioned (Huang *et al.*, 1982). Chelation could explain the abnormal chromosome condensation since metal ions (eg $Ca^{2+}$) are known to be involved in the complex chromosome condensation process. Alternatively the razoxane/histone association, demonstrated by Livingstone *et al.* (1972), could also prevent normal chromosome condensation by disrupting the DNA chain as it begins to condense prior to mitosis. Chromosome condensation occurs at the $G_2$/M stage of the cell cycle, the stage at which razoxane exerts its maximal cytotoxic effect (Sharpe *et al.*, 1970; Creighton, 1974) and from these observations the mode of action appears to be specific to condensing chromatin of eukaryotes (NB prokaryotes do not undergo a DNA condensation cycle).

Razoxane was also shown to affect cell division as seen by the increase in polyploid cells. Similar effects have been demonstrated *in vitro* using time-lapse photography and cytofluorographic analysis of razoxane treated L cells by Creighton (1979). The time-lapse photography showed abnormal cell division, i.e. as the daughter cells moved apart they often remained linked by strands of nuclear material. Eventually, the daughter cells rejoined to form a tetraploid cell or separated with uneven amounts of chromatin. The cytofluorographic analysis of the L-cells showed an increase in cellular DNA content after treatment with razoxane.

The mechanism of action of razoxane is under further investigation using closely related active and inactive analogues of razoxane (Herman *et al.*, 1982) in cultured human lymphocytes.

We would like to acknowledge the statistical help provided by Dr R.A. Ferguson and the technical support of Miss Cheryl Rickard, I.J. Thompson and S. Moore. We would also like to thank Dr T.C. Orton, Dr J.C. Topham and Dr A. Creighton for helpful criticisms and discussions.

### References

AMES, B.N., McCANN, J. & YAMASAKI, E. (1975). Methods for detecting carcinogens and mutagens with the Salmonella/mammalian microsome mutagenicity test. *Mutat. Res.*, **31**, 347.

BAKOWSKI, M.T. (1976). ICRF 159 (±) 1,2-bis(3,5-dioxopiperazin-1-yl) propane NSC-129943; Razoxane. *Cancer Treat. Rev.*, **3**, 95.

CREIGHTON, A.M., HELLMANN, K. & WHITECROSS, S. (1969). Antitumour activity in a series of bisdiketopiperazines. *Nature*, **222**, 384.

CREIGHTON, A.M. (1970). Bisdiketopiperazines: A new class of antitumour agent. In: *Progress in Antimicrobial Anticancer Chemotherapy*, Umezawa, (ed) vol. 1 p. 167. University Park Press: Baltimore.

CREIGHTON, A.M. (1974). The effects of ICRF 159 on sychronised L-cells at various stages of the cell cycle, *Abstracts XIth International Cancer Congress, Vol. 3,* Florence, Italv, p. 423, UICC.

CREIGHTON, A.M. (1979). Mechanism of action of ICRF 159. In *Advances in Medical Oncology, Research and Education* Vol. V. *Basis for Cancer Therapy*, Fox, M. (ed) p. 83. Pergamon Press: Oxford.

DAWSON, (1975). Studies on the stability and cellular distribution of dioxopiperazines in cultured BKH-215 cells. *Biochem. Pharmacol.*, **24**, 2249.

HERMAN, E.H., WITIAK, D.T. HELLMANN, K. & WARAVDEKAR, V.S. (1982). Biological properties of ICRF 159 and related bis(dioxopiperazine) compounds. *Adv. Pharmacol. Chemother.* **19**, 249.

HUANG, Z.X., MAY, P.M., QUINLAN, K.M., WILLIAMS, D.R. & CREIGHTON, A.M. (1982). Metal binding by pharmaceuticals. Part 2. Interactions of Ca(II), Fe(II), Mg(II), Mn(II) and Zn(II) with the intracellular hydrolysis product of the antitumour agent ICRF 159 and its inactive homologue ICRF 192. *Agents and Actions*, **12**, 4.

LIVINGSTONE, D.C. CREIGHTON, A.M. & FISHER, S.W. (1972). Biochemical studies relating to the mechanism of action of the antitumour agent ICRF 159 and related compounds. In *Advances in Antimicrobial and Antineoplastic Chemotherapy.*, Semonsky *et al.* (eds) p. 109, Urban and Schwarzenberg: Munchen.

McCANN, J., CHOI, E., YAMASAKI, E. & AMES, B.N. (1975). Detection of carcinogens as mutagens in the Salmonella/microsome test: Assay of 300 chemicals. *Proc. Natl Acad. Sci.*, **72**, 5133.

SCHMID, W., ARAKAKI, D.T., BRESLAM, N.A. & CULBERTSON, J.C. (1971). The Chinese hamster bone marrow as an *in vivo* test system. *Humangenetik*, **11**, 103.

SHARPE, H.B.A., FIELD, E.O. & HELLMANN, K. (1970). Mode of action of the cytostatic agent 'ICRF 159'. *Nature*, **226**, 524.

WITIAK, D.T., LEE, H.J., HART, R.W. & GIBSON, R.E. (1977). Study of trans cyclopropylbi(diketopiperazine) and chelating agents related to ICRF 159. Cytotoxicity mutagenicity and effects on scheduled and unscheduled DNA synthesis. *J. Med. Chem.*, **20**, 630.